# Impact of Next-Generation Sequencing on Surgical Decision-Making in Branch-Duct IPMN

**DOI:** 10.1245/s10434-026-20023-6

**Published:** 2026-06-24

**Authors:** Maxime Remy, Jeremie Albouys, David Jérémie Birnbaum, Sebastien Gaujoux, Anne Laure Védie, Clémence Descourvières, Jean Michel Gonzalez, Diane Lorenzo

**Affiliations:** 1 Gastroenterology and Endoscopy Unit, Hôpital Nord—AP-HM, Aix-Marseille université, Marseille, France; 2 Gastroenterology and Endoscopy Unit, Hôpital de Limoges, Limoges, France; 3 Digestive Surgery, Hôpital Nord—AP-HM, Aix-Marseille université, Marseille, France; 4 Digestive Surgery, Hôpital La Pitié Salpêtrière- APHP—Sorbonne Université, Paris, France; 5 Gastroenterology and Pancreatology Unit, Hôpital Beaujon, Université Paris Cité, Clichy-La-Garenne, France

**Keywords:** Intraductal papillary mucinous neoplasm (IPMN), Next-generation sequencing (NGS), Therapeutic decision-making, Inter-physician agreement, Fleiss’ kappa

## Abstract

**Background:**

Managing branch-duct intraductal papillary mucinous neoplasms (BD-IPMNs) remains challenging, as guideline criteria (worrisome features/high-risk stigmata) and nomograms often leave uncertainty. Next-generation sequencing (NGS) may provide actionable molecular information that influences clinical decision-making.

**Methods:**

We conducted a national, vignette-based decision-impact study involving 132 clinicians (gastroenterologists and surgeons). Four complex BD-IPMN vignettes (each with two to four worrisome features) were evaluated twice: pre-NGS and post-NGS after disclosure of either a high-risk mutation (positive) or its absence (negative). The primary endpoint was the within-physician change in management in the NGS-concordant direction (NGS+ surveillance to surgery; NGS− surgery to surveillance) assessed using McNemar’s test and mixed-effects logistic regression. Secondary endpoints included change in decision confidence, inter-physician agreement (Fleiss’ κappa), and practitioner factors.

**Results:**

NGS significantly changed management in all vignettes (all *p* < 0.001): with NGS−, surgical plans shifted to surveillance (43% in distal pancreatectomy; 61% in pancreatoduodenectomy scenarios); with NGS+, surveillance shifted to surgery (90% and 72%, respectively; both *p* < 0.001). In mixed-model, post-NGS reduced surgery in NGS− cases (odd ratio [OR] 0.30, 95%confidence interval [CI] 0.16–0.57) and the post-NGS effect was strongly amplified in NGS+ cases (interaction OR 76.51, 95%CI 26.47–221.13). Population-averaged probabilities of choosing surgery shifted from 20% to 7% (NGS−) and 42% to 94% (NGS+). Decision confidence increased (proportional-odds OR 4.66, 95%CI 3.62–6.02, *p* < 0.0001). Inter-physician agreement rose from *κ* = 0.044 (95%CI 0.033–0.055) pre-NGS to *κ* = 0.590 (95%CI 0.580–0.601) post-NGS (*p* < 2×10^−16^). After NGS, practitioner characteristics no longer explained decision patterns.

**Conclusions:**

In complex BD-IPMN scenarios, NGS significantly influences clinician decision-making and reduces inter-physician variability. These findings support its role as a decision-support tool.

Pancreatic ductal adenocarcinoma (PDAC) is highly lethal, increasing in incidence, and lacks population screening.^1,2^ Among PDAC precursors, pancreatic intraepithelial neoplasia is microscopic and undetectable, while intraductal papillary mucinous neoplasms (IPMNs), the only clinically visible precursors, account for 60–70% of pancreatic cysts and affect 7–10% of adults. However, their evolutionary behavior remains difficult to assess.^3^

Current management of IPMNs mainly relies on identifying indirect clinical, biological and radiological signs that predict high-grade dysplasia (HGD) or invasive carcinoma (IC) in the surgical specimen, as stated in the European and international guidelines. The signs are categorized into two groups: worrisome features (WF) (e.g., cyst ≥ 3 cm, enhancing wall or mural nodule < 5 mm, main pancreatic duct (MPD) 5–9 mm, abrupt duct change with distal atrophy, lymphadenopathy, elevated CA19-9, acute pancreatitis, new-onset diabetes, or accelerated growth) and high-risk stigmata (HRS) (e.g., obstructive jaundice with a head cyst, an enhancing mural nodule ≥ 5 mm, or a main pancreatic duct ≥ 10 mm), as outlined in the latest international consensus guidelines on IPMNs.^4,5^ In surgically fit patients, the presence of any HRS generally triggers resection.^4,5^ When WF accumulate, endoscopic ultrasound (EUS) is recommended, and surgery is commonly considered when multiple WF coexist.^4,5^ Observational data suggest that increasing WF burden correlates with higher HGD rates on resection specimens (e.g., ≥ 3 WF : > 60% HGD).^6^ Recognition of WF and HRS remains necessary because cyst-fluid cytology detects high-grade dysplasia or invasive carcinoma with a sensitivity below 30%.^4^

Several nomograms have been proposed to estimate the probability of HGD/IC in IPMN and to support management decisions; they typically combine selected clinical variables with WF/HRS.^7–12^ However, uptake in routine practice remains limited. Many models are complex and require inputs that are not consistently captured; they often do not include the full set of guideline-defined WF/HRS and omit individual baseline PDAC risk factors (age, sex, smoking, family history) as well as operative risk (90-day mortality was 9% after pancreatoduodenectomy and 5% after distal pancreatectomy).^7–13^ In addition, predicted risks and performance vary substantially across models (AUC between 0.7 and 0.8, with positive predictive value [PPV] around 50%), and most tools return only a probability without explicit therapeutic management (surveillance or surgery), which limits bedside decision-making.^7–12^

In this context, by enabling direct analysis of the genetic alterations involved in neoplastic progression, NGS offers a more sensitive and specific tool to assess the malignant potential of IPMNs. A molecular strategy based on detection of high-risk mutations has been developed over the past two decades through basic research and is now accessible to patients via NGS.^4,14–16^ NGS provides diagnostic value through the detection of KRAS and GNAS mutations, and prognostic value by identifying high-risk mutations, such as TP53, SMAD4, or RNF43.^14–18^ these alterations represent key molecular events in the stepwise progression from low-grade dysplasia to invasive carcinoma, as demonstrated in genomic and clonal evolution studies of IPMNs, providing biological plausibility for their clinical interpretation.^19,20^ Notably, a very recent multi-institutional validation study showed that a combined DNA/RNA NGS classifier (PancreaSeq Genomic Classifier), leveraging transcriptomic alterations, improves pancreatic cyst classification and detection of advanced neoplasia.^21^

These high-risk mutations are strongly associated with HGD/IC and have a PPV of approximately 90%, particularly among patients with at least one WF.^15,16,18,21^ Conversely, the absence of high-risk mutations is consistent with low-grade dysplasia with negative predictive value [NPV] of 85%. Access to NGS represents a major advance for patients, with diagnostic performance for detecting HGD far superior to that of conventional cytology and to prediction of current nomograms predictions.^6–12,15,16^ However, its implementation in routine clinical practice remains limited by several barriers, including its considerable cost, the need for access to specialized platforms, and the expertise of trained molecular biologists.

In this study, we evaluated the impact of NGS on clinicians’ decision-making, through a vignette-based design using standardized fictional clinical scenarios representing complex therapeutic decision-making situations involving BD-IPMNs with WF.

The primary objective was to determine whether NGS results changed the management decision (surveillance or surgery) from the pre-NGS plan. Secondary objectives were to profile practitioner and clinical determinants of decision‐making, quantify NGS-related gains in confidence, and test whether NGS homogenized choices across physicians.

## Methods

### Study Design, Clinical Vignettes, and Data Collection

This was a clinical practice study involving physicians managing IPMNs, specifically pancreatic surgeons and gastroenterologists specializing in pancreatic diseases. An online survey (Google Form) was distributed electronically throughout France and Belgium via professional mailing lists.

**Fig. 1:**
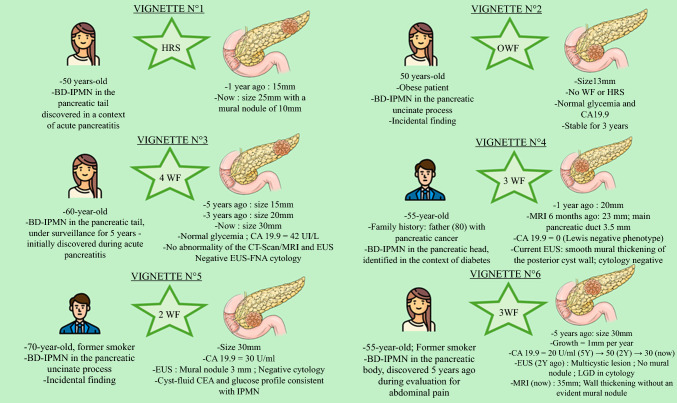
Clinical vignettes of the study

The study was based on six clinical vignettes (one patient per vignette, see Fig. 1). Only BD-IPMNs were included in the vignettes, as these are the only subtypes that can be punctured by EUS-FNA. The first two vignettes represented straightforward, guideline-consistent scenarios (one requiring surgery owing to high-risk stigmata and one requiring surveillance in the absence of risk features) and were used as screening cases. Participants providing at least one response inconsistent with these recommendations were excluded on the basis of pre-specified criteria. The next four vignettes involved complex scenarios of BD-IPMN scenarios with WF to assess practitioners’ decision-making first without NGS and then with NGS results. All participants received the same four vignettes, and for each case the NGS result was identical across respondents.

Each vignette provided information on the patient’s age, comorbidities, symptoms, and imaging findings (including the location of BD-IPMN in the pancreatic head or tail), the presence of WF (two to four per patient) and incorporated individual PDAC risk factors. Because perioperative risk differs significantly between procedures, the vignette set intentionally spanned the management spectrum: one head lesion implying pancreatoduodenectomy, one tail lesion implying distal pancreatectomy, and two surveillance-oriented cases (head and tail, respectively).

For each vignette, participants were asked—without the ability to revise previous responses—to: make an initial management decision (surveillance or surgery), indicate their level of confidence using a 5-point Likert scale, revise their decision after the NGS result was revealed (presence or absence of high-risk mutations), and re-evaluate their confidence level using the same Likert scale.

In addition, sociodemographic and professional data about practitioners were collected: sex, age, surgeon or gastroenterologist, professional status (university professor, hospital-based practice, private practice, or mixed), years of EUS experience for gastroenterologists, number of pancreatic surgeries performed annually and per center for surgeons, and participants’ self-reported familiarity with recent NGS studies in IPMNs. No missing data were observed among the included participants, as completion of all vignette responses was required prior to submission.

### Study Endpoints

The primary endpoint was defined as a change in therapeutic decision following disclosure of NGS results, specifically, a positive NGS result leading to a shift from surveillance to surgery, or a negative NGS result prompting a shift from surgery to surveillance.

Secondary endpoints included: (i) practitioner characteristics associated with the initial decision; (ii) clinical profiles most influenced by NGS; (iii) confidence endpoint—change in clinician confidence from pre- to post-NGS, including whether NGS reinforced an unchanged decision; and (iv) inter-physician agreement before versus after NGS. Our a priori hypothesis was that molecular information would homogenize decisions across physicians. The study was not designed to evaluate diagnostic accuracy or patient-level outcomes.

### Statistical Analysis

Analyses were conducted using IBM SPSS Statistics, version 27 (IBM Corp., Armonk, NY) and R, version 4.4.1 (R Foundation for Statistical Computing, Vienna, Austria).

Categorical variables are presented as percentages and continuous variables as mean ± standard deviations (SD). The primary endpoint—change in management after NGS disclosure (surveillance versus surgery)—was first compared pre- versus post-NGS using McNemar’s test, then modeled with a mixed-effects logistic regression including fixed effects for time (pre/post), NGS result (negative/positive), and their interaction, with random intercepts for physician and vignette to account for clustering. Results are given as odds ratios (ORs) with 95% confidence intervals (CIs), and marginal predicted probabilities were calculated for interpretability.

Inter-physician agreement was evaluated pre- and post-NGS using Fleiss’ κappa (95% CI, two-sided *p* values), with the change in agreement (Δκ) assessed by paired bootstrap resampling; overall and specific (positive/negative) agreements were also summarized.

Decision confidence (5-point Likert scale) was analyzed by comparing pre- and post-NGS ratings within each vignette using paired Wilcoxon signed-rank tests, with Holm correction across vignettes. Practitioner-level associations for secondary questions were explored with standard univariable tests (chi-squared for categorical variable, *t*-test for continuous variables). Two-sided *p* < 0.05 was considered statistically significant.

## Results

### Practitioner Characteristics

**Fig. 2:**
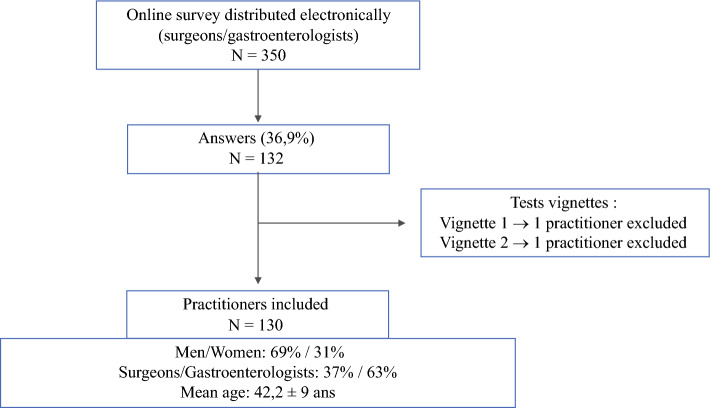
Flow chart of the survey process

Among the 352 clinicians contacted, 132 (37%) completed the survey and 2 were excluded based on responses to screening vignettes P1 and P2 (see Fig. 2: flow chart); leaving 130 participants, of whom 63% (*n* = 82) were gastroenterologists and 37% (*n* = 48) were pancreatic surgeons, with a mean age of 42 ± 9.0 years; 69% (*n* = 90) were men. Regarding practice setting, 60% (*n* = 78) were affiliated with university hospitals (35% without a PhD, 25% with a PhD), 21% (*n* = 27) worked in private practice, 11% (*n* = 14) were based in nonuniversity public hospitals, and 8% (*n* = 11) had mixed activity (both hospital and private). Among gastroenterologists, 86% (72/82) performed EUS. Among surgeons, annual center volume was > 15 pancreatectomies (high-volume) for 66%, 5–15 for 23%, and < 5 (low-volume) for 11%. Mean professional experience was reported as 9.8 ± 8.3 years of EUS experience for gastroenterologists and 13 ± 8.4 years of pancreatic surgery experience for surgeons.

Easy access to a NGS platform was reported by 24% (*n* = 31) and prior familiarity with NGS in BD-IPMN literature by 49% (*n* = 64).

### Initial Therapeutic Decisions (pre-NGS) and Associated Factors

In vignette P3 (woman, pancreatic tail, 4 WF), surgery was selected by 46% (*n* = 60) of clinicians. In vignette P4 (man, head located BD-IPMN, 3 WF), surgery was chosen in 18% (*n* = 23) of cases. In vignette P5 (man, uncinate process BD-IPMN, 2 WF), 42% of respondents (*n* = 54) opted for surgery. Finally, in vignette P6 (woman, BD-IPMN in the pancreatic body, 3 WF), surgery was proposed in 34% (*n* = 44) of cases (Table 1).

**Table 1:** Impact of NGS on therapeutic decisions across four complex clinical vignettes

Vignette	IPMN location	No. of worrisome features	Initial decision: surveillance	Initial decision: surgery	NGS result (High-Risk Mutation)	Final decision: surveillance	Final decision: surgery	No. of clinicians who changed decision	Main direction of change	*p*-value (McNemar)
P3 (female)	Tail	4	70 (54%)	60 (46%)	*TP53* mutation	7 (5%)	123 (95%)	63 (90%)	Surveillance → Surgery	<0.001
P4 (male)	Head	3	107 (82%)	23 (18%)	No mutation	121 (93%)	9 (7%)	14 (61%)	Surgery → Surveillance	<0.001
P5 (male)	Uncinate	2	76 (58%)	54 (42%)	*SMAD4* mutation	21 (16%)	109 (84%)	55 (72%)	Surveillance → Surgery	<0.001
P6 (female)	Body	3	86 (66%)	44 (34%)	No mutation	105 (81%)	25 (19%)	19 (43%)	Surgery → Surveillance	<0.001

This table summarizes the clinical characteristics of each vignette (location of the IPMN, number of worrisome features), initial management decisions (surgery vs. surveillance), NGS results (presence or absence of a high-risk mutation), and final decisions after NGS. It also reports the number of clinicians who changed their decision, the main direction of change, and the statistical significance (McNemar test). NGS significantly influenced therapeutic decisions by promoting surgery in cases with high-risk mutations and favoring surveillance in their absence

Across vignettes, the pre-NGS choice (surveillance versus surgery) was associated was associated with practitioner factors, though inconsistently: clinicians who chose surveillance tended to be older and to have longer EUS experience (*p* = 0.02–0.04), while not performing EUS or working in nonuniversity hospitals was associated with choosing surgery (*p* = 0.002 and *p* = 0.039). In scenarios involving distal pancreatectomy, clinicians from high-volume centers (> 15 pancreatectomies/year) were more likely to favor surveillance (*p* = 0.008). No differences were observed between gastroenterologists and surgeons.

### ***Primary Endpoint (***Table 1***)***

Table 1 presents the initial management decisions based on BD-IPMN location and number of WF, as well as the change in management following NGS results for each vignette.

For all four vignettes, NGS disclosure significantly changed management (McNemar, *p* < 0.001). A high-risk mutation increased the switch from surveillance to surgery (90% in distal pancreatectomy scenarios and 72% in pancreaticoduodenectomy scenarios) (both *p* < 0.001). Conversely, negative NGS results shifted initial surgical choices toward surveillance (43% in distal pancreatectomy and 61% in pancreaticoduodenectomy) (both *p* < 0.001).

In the mixed-effects logistic regression accounting for clustering by physician and vignette (*N* = 1040 observations; 130 physicians; 4 vignettes), post-NGS results reduced the odds of surgery in NGS-negative scenarios (OR 0.30, 95%CI 0.16–0.57; *p* < 0.001). At baseline (pre-NGS), NGS-positive vignettes had higher odds of surgery than NGS-negative ones (OR 2.83, 95%CI 1.12–7.14; *p* = 0.027). Notably, the post-NGS effect was strongly amplified in NGS-positive scenarios (interaction OR 76.51, 95%CI 26.47–221.13; *p* < 0.001).

**Fig. 3:**
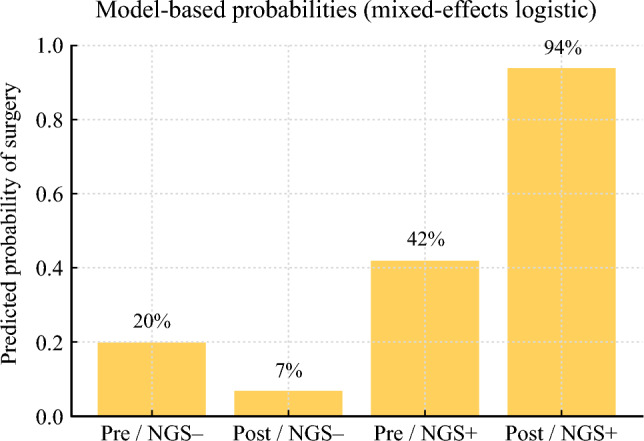
Model-based probabilities of surgery before and after disclosure of NGS results *NGS* next-generation sequencing

For interpretability, fixed-effect predicted probabilities of surgery were approximately 20% pre-NGS versus 7% post-NGS when NGS was negative (absolute difference −13%), and 42% pre-NGS versus 94% post-NGS when NGS was positive (absolute difference +52%) (Fig. 3).

### Secondary Endpoints

#### ***Impact of NGS on Reinforcement of the Initial Therapeutic Decision (***table 2***)***

**Table 2:** Impact of NGS on clinicians’ confidence in therapeutic decision-making

Vignette	IPMN location	Mean confidence score before NGS	Median confidence score before NGS	NGS result	Mean confidence score after NGS	Median confidence score after NGS	Δ confidence	*p*-value (Wilcoxon)
P3 (female)	Tail	3.16 ± 1.06	*3 (IQR : 3-4)*	*TP53* mutation	3.92 ± 0.99	*4 (IQR : 3-5)*	+0.76	<0.001
P4 (male)	Head	3.08 ± 0.95	*3 (IQR : 3-4)*	No high-risk mutation	3.71 ± 1.05	*4 (IQR : 3-4)*	+0.63	<0.001
P5 (male)	Uncinate	3.45 ± 0.97	*4 (IQR : 3-4)*	*SMAD4* mutation	3.87 ± 0.87	*4 (IQR : 3-4)*	+0.42	<0.001
P6 (female)	Body	3.22 ± 0.87	*3 (IQR : 3-4)*	No high-risk mutation	3.78 ± 0.83	*4 (IQR : 3-4)*	+0.56	<0.001

This table shows, for each vignette, the mean confidence score before and after receiving NGS results (measured on a 5-point Likert scale), the associated NGS result (mutation or no high-risk mutation detected), the average change in confidence score (mean and median), and statistical significance (paired Wilcoxon test). In all scenarios, NGS significantly increased the clinicians’ confidence in their chosen management strategy. Decision confidence increased significantly after NGS disclosure (proportional-odds OR 4.66, 95%CI[3.62–6.02], *p*<0.0001)

When management remained unchanged, NGS reinforced the initial choice: Likert confidence increased significantly in all four vignettes (Wilcoxon signed-rank, *p* < 0.001 for each). Positive NGS increased confidence in surgery (P3, P5), while negative NGS increased confidence in surveillance (P4, P6).

#### Factors Associated with Therapeutic Decisions after NGS

No sociodemographic or professional variable was significantly associated with a change in decision following NGS, supporting the idea that NGS guided clinical choices independently of practitioner background or expertise.

#### *Inter-physician agreement (*Fig. 4*)*

**Fig. 4:**
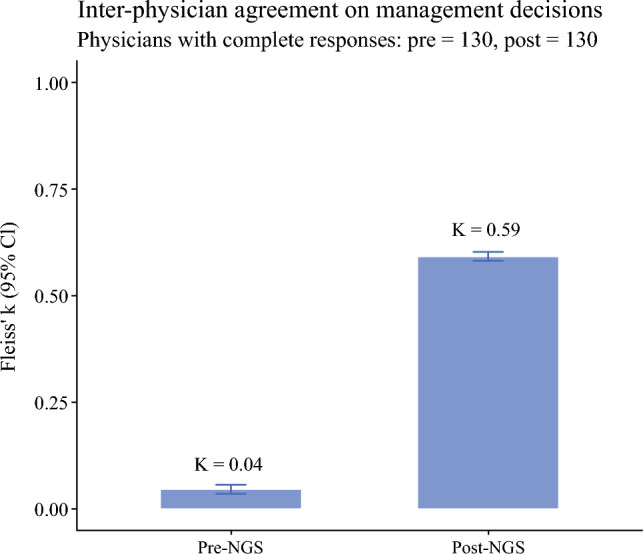
Impact of NGS results on inter-physician agreement for management decisions *NGS* next-generation sequencing

Among the 130 physicians with complete responses, inter-rater agreement (Fleiss’ κappa) increased from 0.044 (95%CI: 0.033–0.055) (slight) before NGS disclosure to 0.590 (95%CI: 0.580–0.601) (moderate) after. The paired bootstrap change was Δκ = 0.544 (95% CI 0.465–0.618; *p* < 2 × 10^−16^). Overall raw agreement, the proportion of identical management choices across physicians, regardless of category, increased from 56.6% pre-NGS to 79.5% post-NGS. In other words, following NGS disclosure, almost 80% of decisions were concordant, whereas this was just over 50% prior to disclosure.

## Discussion

In this national study including 130 clinicians (gastroenterologists and pancreatic surgeons), access to NGS shifted management of BD-IPMN toward NGS-concordant decisions: surgical indications increased when high-risk mutations were present and decreased when they were absent, in scenarios involving either pancreatoduodenectomy or distal pancreatectomy. NGS also increased decision confidence in these complex situations. Notably, practitioner-level factors that influenced pre-NGS choices (age, EUS practice, center volume) no longer explained post-NGS decisions, suggesting a homogenizing effect of molecular information. This was further reflected by a marked increase in inter-physician agreement (Fleiss’ κ from 0.04 to 0.59).

Deciding on surgery for IPMNs is inherently difficult: treatment should detect HGD or early cancer while sparing patients with LGD from high-morbidity pancreatic resections, such as postoperative fistula, diabetes.^4,6,13,22^ Recognition of HRS and WF helps identify patients at increased risk of advanced neoplasia. In the absence of both, the risk of HGD/IC is under 5%.^4,10^ However, the presence of WF alone does not reliably predict advanced disease, with PPV ranging from 29% to 52%.^7,10,22–24^ Several nomograms have been developed in this context to refine risk stratification, but they remain poorly applicable.^7–12^ In our study, for vignette 3, predicted risk of HGD/IC ranged from 7% to 37% across nomograms, yet 46% of clinicians still chose immediate surgery. Reflecting this lack of consensus, pre-NGS decisions were essentially indistinguishable from chance agreement (Fleiss’ *κ* = 0.04). Real-world evaluations suggest only a modest clinical impact, with about a 15% reduction in unnecessary surgery.^8,11^ Their limited impact likely reflects a mismatch with real-world decision-making: bedside choices integrate individual risk of pancreatic adenocarcinoma, clinical warning signs (weight loss, pain), the full WF/HRS profile (often only partially encoded), and procedural risk. Importantly, operative morbidity is not uniform (distal pancreatectomy is less morbid than pancreaticoduodenectomy yet current tools do not incorporate these elements).

In contrast to indirect risk surrogates, cyst-fluid NGS provides a direct genomic readout of the lesion, identifying high-risk mutations (e.g., TP53, SMAD4, RNF43) causally linked to high-grade dysplasia and invasion; their absence is consistent with LGD biology. Performance is high, with PPV and NPV typically reported in the range of 80–90% in selected cohorts, particularly in WF-positive BD-IPMN.^15,16,18,21^ However, negative predictive value is not absolute, and residual risk of occult high-grade dysplasia or invasive carcinoma persists. This likely explains why, in our study, a proportion of clinicians continued to recommend surgery in NGS− scenarios, particularly when multiple worrisome features were present. Rather than reflecting disregard of molecular data, this pattern may indicate that clinicians integrate NGS findings within a broader clinical framework, particularly in anatomically lower-morbidity procedures such as distal pancreatectomy. The strong genotype/phenotype correlation reported in IPMNs likely underlies clinicians’ responsiveness to molecular results, while the imperfect sensitivity of current platforms may account for cautious decision-making in high-risk contexts.^15,16,18,21^ In real-world settings, a recent study showed that NGS findings led to a change in strategy in 26% of patients, most often resulting in reduced intensity or discontinuation of surveillance.^25^ Our objective was not to demonstrate diagnostic accuracy of NGS, but to evaluate how clinicians incorporate molecular information into their reasoning. In complex BD-IPMN scenarios, clinicians were highly responsive to molecular information: NGS-positive findings shifted choices toward surgery, NGS-negative results toward surveillance; decision confidence increased; and inter-physician variability decreased markedly, with agreement rising from *κ* = 0.04 pre-NGS to *κ* = 0.59 post-NGS (Δ*κ* = 0.54). Switching appeared more pronounced in NGS-positive than NGS-negative scenarios; however, this comparison should be interpreted cautiously given the vignette-level assignment of NGS polarity. Increased agreement following NGS disclosure should not be equated with correctness. Decision-impact studies measure convergence in clinician recommendations, not patient-level outcomes. Whether this alignment reflects improved decision quality or over-reliance on molecular data cannot be determined within the present design and requires prospective validation. Here, we cannot determine whether greater confidence reflects improved calibration or over-reliance on molecular information. Finally, the large interaction OR (76.51) likely reflects the underlying probability scale, with low baseline surgery rates in NGS-negative scenarios and near-saturation in NGS-positive scenarios after NGS disclosure, a context in which odds ratios may overstate proportional effects.

Decision-impact studies of genomic assays in other cancers have shown similar effects on clinician decision-making and concordance.^26–28^ These parallels suggest that molecular data can shift individual decisions and contribute to standardizing practice across clinicians.

Our study has several strengths. We enrolled a large, diverse cohort of 130 clinicians (gastroenterologists and pancreatic surgeons) from various settings. The within-physician pre/post design directly captures behavioral change attributable to NGS. The vignettes focused on ambiguous, real-world BD-IPMN scenarios where molecular results are relevant to decision making. In addition, the changes in management, we assessed decision confidence and inter-physician agreement, demonstrating that NGS can recalibrate choices and standardize practice. The study addresses a timely, innovative topic with an original decision-impact perspective. However, this work also has limitations. The 37.5% response rate may introduce selection bias. As in most survey-based studies, respondents may differ systematically from nonrespondents. Clinicians with greater involvement in pancreatic cyst management or prior exposure to molecular testing may have been more likely to participate, potentially enriching the cohort with practitioners more familiar with or receptive to NGS. Conversely, non-responders may include clinicians practicing in lower-volume or resource-limited settings, or those less confident in interpreting molecular results. Because detailed data on nonrespondents were not available, we cannot formally assess these differences. Therefore, our findings may preferentially reflect the perspectives of clinicians engaged in pancreatic disease management and may not be fully generalizable to all practice environments. Cost-effectiveness, access, and turnaround time were not evaluated. Notably, only 24% of respondents reported easy access to NGS, reflecting its uneven availability. Although molecular testing may improve risk stratification and help avoid unnecessary surgery, broader implementation raises important questions regarding equitable access and reimbursement. Prospective studies should therefore integrate economic and access considerations alongside clinical outcomes. All cases involved BD-IPMN, limiting extrapolation to main-duct or mixed forms. Another important limitation of our study lies in the intentionally simplified molecular framework used in the vignettes. NGS results were presented as a binary signal centered on the presence of canonical high-risk alterations (TP53 or SMAD4) versus absence of such mutations. While this approach allowed us to isolate the directional decision impact of a clear molecular cue, real-world cyst NGS reports are frequently more complex. Clinicians often face intermediate-risk alterations, low allele fraction detection of TP53 or SMAD4, variants of uncertain significance, technical failures, or mixed molecular profiles. In addition, IPMNs are characterized by spatial and clonal heterogeneity, and cyst fluid or focal sampling may not fully represent the most dysplastic epithelial component. In such settings, interpretation may be more nuanced and less deterministic than in our experimental model. Therefore, our findings reflect the impact of a simplified high-risk/low-risk molecular dichotomy and may not fully capture the complexity of real-world molecular reporting.

In conclusion, this national vignette-based study demonstrates that clinicians integrate NGS information into BD-IPMN decision-making: molecular results significantly influenced recommendations, increased decision confidence, and improved inter-physician agreement. These findings suggest that NGS may function as a decision-support tool in complex scenarios. Prospective real-world studies incorporating standardized panels, defined interpretive thresholds, and patient-level outcomes are required to determine whether these molecularly informed strategies translate into improved clinical results.
